# Intestinal Epithelial Cell Exosome Launches IL-1β-Mediated Neuron Injury in Sepsis-Associated Encephalopathy

**DOI:** 10.3389/fcimb.2021.783049

**Published:** 2022-01-17

**Authors:** Shaosong Xi, Yunguang Wang, Chenghao Wu, Weihua Peng, Ying Zhu, Wei Hu

**Affiliations:** Department of Critical Care Medicine, Affiliated Hangzhou First People’s Hospital, Zhejiang University School of Medicine, Hangzhou, China

**Keywords:** intestinal epithelial cell, exosome, sepsis-associated encephalopathy, fecal microbiota transplantation, IL-1β, mesenteric lymph nodes

## Abstract

**Background:**

Gut–microbiota–brain axis links the relationship between intestinal microbiota and sepsis-associated encephalopathy (SAE). However, the key mediators between them remain unclear.

**Methods:**

Memory test was determined by Water maze. Intestinal flora was measured by 16S RNA sequencing. Neurotransmitter was detected by high-performance liquid chromatography (HPLC). Histopathology was determined by H&E, immunofluorescence (IF), and terminal-deoxynucleoitidyl transferase mediated nick end labeling (TUNEL) staining. Flow cytometry was employed to determine the proportion of macrophages.

**Results:**

Fecal microbiota transplantation (FMT) relieved hippocampus impairment of SAE rats by inhibiting inflammation cytokine secretion, the expression of IBA-1 and neurotransmitter disturbance, and cell apoptosis and autophagy, accompanied by the reduced M1 polarization and M1 pro-inflammation factors produced by macrophages in mesenteric lymph nodes (MLNs). Actually, M1 polarization in SAE rats depended on intestinal epithelial cell (IEC)-derived exosome. GW4869-initiated inhibition of exosome secretion notably abolished M1 polarization and the secretion of IL-1β. However, GW4869-mediated improvement of hippocampus impairment was counteracted by the delivery of recombinant interleukin (IL)-1β to hippocampus. Mechanistically, IEC-derived exosome induced the excessive circulating IL-1β produced by CP-R048 macrophages, which subsequently induced damage and apoptosis of hippocampal neurons H19-7 in an autophagy-dependent manner. And reactivation of autophagy facilitates intestinal IL-1β-mediated hippocampal neuron injury.

**Conclusion:**

Collectively, intestinal flora disturbance induced the exosome release of IECs, which subsequently caused M1 polarization in MLNs and the accumulation of circulating IL-1β. Circulating IL-1β promoted the damage and apoptosis of neurons in an autophagy-dependent manner. Possibly, targeting intestinal flora or IEC-derived exosome contributes to the treatment of SAE.

## Introduction

Sepsis is a fatal organ dysfunction caused by host response imbalance induced by infection. Sepsis and its related complications are the main cause of death in critically ill patients (Singer et al., 2016). Sepsis-associated encephalopathy (SAE) is one of the common complications and the most common encephalopathy in patients of sepsis relating to poor prognosis and long-term cognitive dysfunction (Gofton and Young, 2012). It is reported that many factors are involved in the pathogenesis of SAE, including inflammatory cytokines, blood–brain barrier breakdown, ischemic process, neurotransmitter changes, and mitochondrial dysfunction, but the specific mechanism has not been figured out (Adam et al., 2013). It is reported that uncontrolled neuroinflammation and ischemic injury are closely relevant to the development of brain injury, which are common manifestations in patients with severe sepsis (Ren et al., 2020). Its pathogenesis has not yet been clarified, and there is no specific medicine or therapeutic approach that effectively cures sepsis (Zhu and Zhou, 2018; Ehler et al., 2019). Future research needs to clarify the key points of pathophysiological damage such as brain inflammatory response, blood–brain barrier, and abnormal neurotransmitter transmission preventing permanent damage to the brain function of SAE patients.

The intestinal flora participates in the regulation of the host’s neuro–endocrine–immune network and realizes the signal transmission between the brain and the intestine through neuroendocrine and autonomic nerves (that is, brain–intestine interaction) (Mayer et al., 2015). A large number of studies have pointed out that the intestinal flora is disordered and related to the formation of various brain diseases, such as Alzheimer’s disease, spectrum disorder, and Parkinson’s disease (Sampson et al., 2016; Harach et al., 2017; Luna et al., 2017; Shen et al., 2017). It is reported that the alteration of intestinal flora can occur in the early stage, which affects the severity of SAE through the vagus nerve in patients with sepsis (Li et al., 2018). Moreover, bacterial translocation and gut-origin sepsis are proven to participate in various clinic systemic infectious complications and organ dysfunctions (Runkel et al., 1991; Deitch, 2012). The evidence suggests that intestinal flora dysfunction may be one of the important inducements to SAE. Actually, in the gastrointestinal tract, gut microbiota can translocate to mesenteric lymph nodes (MLNs) (Berg and Garlington, 1979), accompanied by the activation of inflammatory cells, leading to an increase in the number of macrophages. Bacterial translocation and the subsequent inflammatory compounds are the cause of sepsis, which can be determined directly by culture of MLNs (Vaishnavi, 2013). Therefore, bacterial flora disorder-mediated immune response in MLN was involved in the development of sepsis. However, the relationship between SAE and MLN has not been reported.

Intestinal epithelial cells (IECs) interconnect the local immune system and the gut microbiome and are essential for maintaining the health of the host. MLNs are an important part of the immune system, which is reported to participate in the induction of local immune responses to luminal antigen by mediating antigen-presenting cells/T cell interactions and by releasing cytokines *via* activating T cells (Fay et al., 2017). The intestinal flora directly activates the development and differentiation of intestinal epithelium and plays an important role in maintaining the integrity of IECs (Okumura and Takeda, 2017). IECs exert immunomodulatory effects by affecting intestinal mucosal immune cells, including innate lymphoid cells (ILCs) and macrophages (Jaensson et al., 2008). It is speculated that the intestinal flora may regulate the local immune response in MLNs through IECs, which subsequently affects the progression of SAE, but the specific mechanism needs to be explored in depth.

Herein, we hypothesized that bacterial flora disorder mediated the local immune response in MLNs possibly through regulating IECs. The activation of the immune response might subsequently induce the increased circulating inflammatory factors, resulting in the progression of SAE. The present study was aimed to confirm the hypothesis. Possibly, our results may provide evidence for treating prospects of gut–brain signal axis.

## Materials and Methods

### Animals

Eight-week-old male Wistar rats were purchased from China Three Gorges University (SCXK-2017-0012). All animals were housed in a specified pathogen-free (SPF) experimental animal center at 20°C ± 2°C, with a 12-h dark–light cycle. All animals had *ad libitum* access to normal chow and water. Animal experiments were carried out with approval by the Ethics Committee of the Affiliated Hangzhou First People’s Hospital and conducted in accordance with the China Code of Practice for the Care and Use of Animals for Scientific Purposes. Cecal ligation and puncture (CLP) was used to induce SAE model, as per previous procedures (Li et al., 2015). All rats were employed to establish the SAE model after a week of acclimatization. In brief, the cecum was exposed using scissors and ligated at half the distance to the end with a 1-0 Prolene thread after sterilization. Subsequently, the cecum was impaled using a 20 G needle, and the intestinal content was allowed to discharge out from cecum. Then, the abdominal membrane and skin were sutured using 100 Nylon lines. The cecum of Sham-operated rats was exposed according to the above procedure, without ligation and puncture and sutured in two layers. After surgery, the model rats were randomly assigned into two groups: SAE rats and fecal microbiota transplantation (FMT)-challenged SAE rats. FMT was conducted as previously described with a few modifications (Ekmekciu et al., 2017). Briefly, fresh feces were collected from 8-week normal and gender-matched Wistar rats. The feces (3–5 g) were homogenized in 5 ml of sterile phosphate buffer saline (PBS) and filtered twice using a sterile metal screen. After 2 days postoperative, 2 ml filtering medium were gavaged into each rat three times daily for 7 consecutive days. The survival rates were 41.67% and 50% in model group and intervention group, respectively. In addition, when the rats could not move on the second postoperative day, the rats were excluded from this study. For the administration of GW4869 (MCE, HY-19363) and interleukin (IL)-1β (Abcam, Ab73589), the GW4869 reagent was given by intraperitoneal injection and the recombinant IL-1β was delivered to the left hippocampus by stereotaxic surgeries 2 days postoperative (Jean et al., 2015). Briefly, rats were anesthetized by isoflurane using an anesthetic machine before surgery. Animal stereotaxic instrument with a microsyringe was used to inject the recombinant IL-1β. After surgery, the pinhole was sealed by dental silicate cement and the skin was sutured. Then, the surgical wounds were disinfected with iodophor. GW4869 is routinely stored at −80°C as a stock suspension in dimethylsulfoxide (DMSO). The GW4869 and IL-1β were diluted in medium for *in vitro* assay and in saline for *in vivo* assay. The tissues were harvested at the end of modeling (9 days after surgery).

### Neurological Deficit Score

Neurological scores were collected for each rat on day 1 and day 7 after surgery. All rats scores were produced as follows: 2 points, no symptoms; 1 point, hyporeflexia; 0 point, reflection loss.

### Morris Water Maze

At the end of modeling (9 days after surgery and before execution), Morris water maze task was performed in a circular pool with a diameter of 150 cm and 50 cm height filled with water (21°C ± 1.5°C) in an isolated environment according to a previous study (Xin et al., 2021). On day 1, all animals were trained, learned, and memorized on a visual platform to exclude mice with dyskinesia. The device was divided into four quadrants, and the rats were required to find the location of a hidden platform, 1 cm below the water surface, for 4 consecutive days on days 2–5 after surgery. Briefly, all rats were allowed to search the platform for a period of 120 s. If rats could find the platform in 2 min, the rats were allowed to rest on the platform for 10 s. The rats would be replaced on the platform for an additional 10 s to strengthen their memory if the rats could not find the platform within 120 s. The assay was used to evaluate the ability of learning and memory from days 2 to 5. On day 6 of the Morris water maze test, spatial probe test was employed to determine their memory retention capability after removing the platform. The moving trail was monitored with a video camera on the top. The escape latency and distance of swimming in the target quadrant were recorded. A completely randomized, double-blind, controlled trial was performed during behavioral tests.

### 16S RNA Sequencing

At the end of the experiment (9 days after surgery), 1.5 g fresh feces of each rat were collected and were batched for microbiome assessment using 16S ribosomal RNA (rRNA) gene V3-V4 region-based sequencing using HiSeq PE150 platform. Briefly, genomic DNA was extracted using DNA stool mini kit (Qiagen, USA). Subsequently, the bacterial 16S rRNA gene (V3-V4 region) was amplified using PCR and analyzed by Novogene Biotechnology Technology (Tianjin, China). The sequencing data to the repository recommended at GenBank (Accession number: PRJNA767832; SUB10460014: PRJNA767832, https://www.ncbi.nlm.nih.gov/bioproject/PRJNA767832).

### ELISA

After modeling (9 days after surgery), the whole blood and hippocampus tissues were harvested. After 2 h of standing, the whole blood was centrifuged for 3 min at 9,000 rpm. The serum was then collected to analyze the expression of cytokines. The hippocampus tissues were homogenized, and the levels of IL-1β (Elabscience, E-EL-R0012c), IL-6 (Elabscience, E-EL-R0015c), IL-12 (Elabscience, E-EL-R0064c), tumor necrosis factor (TNF)-α (Elabscience, E-EL-R2856c), IL-18 (Elabscience, E-EL-R0567c), colony-stimulating factor (CSF)-1 (Elabscience, E-EL-R0601c), IL-4 (Elabscience, E-EL-R0014c), IL-10 (Elabscience, E-EL-R0016c), transforming growth factor (TGF)-β (Elabscience, E-EL-0162c), and IL-13 (Elabscience, E-EL-R0563c) were determined according to the manufacturers’ instructions.

### Immunofluorescence

In brief, paraffin sections of the hippocampus tissues (collected 9 days after surgery) were dewaxed and rehydrated. The slices were incubated in citrate buffer to retrieve antigen and were then blocked with 1% bovine serum albumin (Sigma-Aldrich, St. Louis, USA) for 1 h. Subsequently, the sections were incubated with the primary antibody against IBA-1 (1:100, Abcam, Ab178847) at 4°C overnight and lastly in Cy3-labeled secondary antibody at room temperature for 1 h. The nucleus was stained with 4-6-diamidino-2-phenylindole (DAPI) (Beyotime, C1002, China). The slices were dehydrated, mounted using Fluoromount-GTM (SouthernBiotech, 0100-01 Birmingham, USA), and photographed under the inverted fluorescence microscope (Leica, Wetzlar, Germany).

### High-Performance Liquid Chromatography

Briefly, the homogenized hippocampus tissues (collected 9 days after surgery) were diluted 10 times by mobile phase. Then, 64-μl samples were mixed with 32 μl ortho-phthalaldehyde (OPA) and 1,040 μl sodium tetraborate solution (pH 9.18). After 3 min of standing, the mixture was detected by high-performance liquid chromatography (HPLC) (Agilent, 1200). According to the standard curves of glutamate (Glu) and γ-aminobutyric acid (GABA), the concentrations of Glu and GABA were calculated based on their peak areas.

### TUNEL

Hippocampus tissues (collected 9 days after surgery) was fixed with 4% paraformaldehyde for more than 24 h and conducted to paraffin embedding. The paraffin-embedded tissues were deparaffinized with xylene, hydrated with graded ethanol, and pretreated with proteinase K. After blocking the endogenous peroxidase using 3% H_2_O_2_, the slices were incubated in terminal deoxynucleotidyl transferase (TdT) (Roche, Basel, Switzerland) or tris buffered saline (TBS). The sections were then stained with secondary antibody, restained with DAPI, and counterstained with Fluoromount-GTM. Then, the slices were photographed by fluorescence microscope (Olympus, BX53). The apoptotic rate was represented by the average number of TUNEL-positive cells in each field.

### Western Blotting (WB)

Total protein of *ex vivo* cells, exosomes, and hippocampus tissue were extracted using radioimmunoprecipitation assay (RIPA) solution lysis buffer (Beyotime, Shanghai, China). The concentration was evaluated by bicinchoninic acid (BCA) kits, and then 30 μg protein was separated by sodium dodecyl sulfate-polyacrylamide gel electrophoresis (SDS-PAGE). The separated protein was then transferred to the activated polyvinylidene difluoride (PVDF). The membrane was blocked with 5% non-fat blocking grade milk (Bio-Rad, Hercules, CA, USA) and subsequently incubated with the following primary antibodies against CD81 (Abcam, Ab109201), CD9 (Abcam, Ab92726), CD63 (Affinity, AF5117), CD80 (Abcam, Ab215166), CD68 (Affinity, AF7518), CD206 (Abcam, Ab64693), CD163 (Abcam, Ab182433), EpCAM (Affinity, DF6311), BAX (Cell signaling, 2772), Bcl-2 (Abcam, Ab59348), Caspase 3 (Abcam, Ab13847), and LC3 (Cell signaling, 4108). Then, the membrane was incubated with appropriate horseradish peroxidase-conjugated secondary antibodies for 1 h at room temperature. Subsequently, the blots were visualized by Versa Doc (Bio-Rad Laboratories, Inc.). Glyceraldehyde phosphate dehydrogenase (GAPDH) (AB-P-R 001) served as the internal control.

### H&E Staining

The paraffin-embedded hippocampus tissues (collected 9 days after surgery) were dewaxed, rehydrated, and stained with hematoxylin (Sigma, H9627) for 5 min. After washing for 2 min with running water, the slices were incubated with 1% eosin (Sinopharm Group, 71014544) for 5 min and then placed into purified water for 30 s. Subsequently, the sections were placed in graded ethanol for hydration and dimethylbenzene for vitrification. After mounting with neutral balsam, the results were observed and photographed under a microscope (Olympus, IX51).

### Flow Cytometry

The exosomes were extracted from IECs of Sham rats, SAE rats, and FMT-treated SAE rats (collected 9 days after surgery). Subsequently, CP-R048 cells treated with lipopolysaccharide (LPS) or a different source of exosome were used to determine the proportion of M1 or M2 macrophages. The CP-R048 cells were harvested and conducted to cell counting. Here, 5 × 10^5^ cells diluted in 1 ml PBS were incubated with antibodies against CD80-PE, CD68-FITC, CD206, and CD163 at 4°C for 30 min. Immunoglobulin G (IgG) served as the isotype control antibody. After transient centrifugation, the pellets were resuspended with 500 μl staining buffer and then analyzed by flow cytometry (BECKMAN, CytoFLEX).

### Intestinal Epithelial Cell Isolation, Exosome Isolation, and Transmission Electron Microscopy

For the isolation of IECs, the colon tissues were harvested from newborn rats, and then the mesentery was removed. After repeatedly cleaning, the colon tissues were cut into 1-mm^3^ fragments that were then digested with collagenase (type I) and hyaluronidase for 25 min at 37°C. Subsequently, the supernatants were collected after 1 min of standing. The supernatants were centrifuged for 5 min at 100 g, and the cell pellets were resuspended using complete medium and cultured in dish precoated with polylysine (Wuhan Procell Life Science and Technology). When the confluence of IECs reached 70%–80%, exosomes were extracted by using MagCapture Exosome Isolation Kit (293-77601, Wako, Japan). The purified exosomes dissolved in PBS were added onto a 2-mm sample-loaded copper mesh. After 1 min of standing, the excess liquid was gently removed using filter paper and the samples were washed gently with distilled water and then air-dried at room temperature. Subsequently, the morphological feature was obtained with transmission electron microscopy (TEM; Hitachi, Japan).

### Nanoparticles Tracking Analysis (NTA)

NTA technology (ZetaView, Particle Metrix, Germany) was used to determine the exosome diameter and the number of exosomes. The scattered light signals of isolated exosomes were collected through an optical microscope, and a section of Brownian motion images was taken in solution. Then, the concentration and diameter of the particles were tracked, analyzed, and calculated.

### Quantitative Real-Time PCR

After stimulation, CP-R048 cells were conducted to total RNA extraction using TRIzol reagent (Roche, USA). Equal RNA was reverse transcribed into cDNA using reverse transcription kit (Takara, Dalina, China). The amplified reaction was performed in a FAST7500 real-time PCR system (ABI, USA). GAPDH was identified as the internal control. All primer sequences were shown in **Table S1**. The relative expression level was calculated as per the 2^-ΔΔCt^ method.

### Statistical Analysis

Data were shown as the means ± SD. IBA-1- and TUNEL-positive cells in immunofluorescence (IF) assay were analyzed by using Image Pro Plus 6.0 software. T-test analysis and one-way ANOVA (Least Significant Difference test) were used to assess the difference between the two groups and between multiple groups, respectively (GraphPad Prism 7.0). P < 0.05 was considered statistically significant.

## Results

### Fecal Microbiota Transplantation Ameliorates Memory Impairment in Sepsis-Associated Encephalopathy Rats

**Figure 1:**
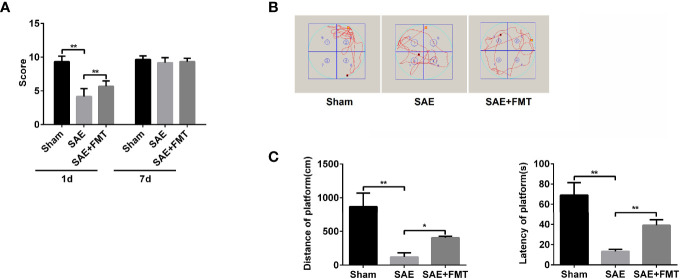
Effects of fecal microbiota transplantation (FMT) on memory impairment of sepsis-associated encephalopathy rats. **(A)** The rats were processed with Sham control and cecal ligation and puncture surgeries. The sepsis-associated encephalopathy (SAE) rats received control reagent or FMT. Then, the neurologic deficit score was measured in the rats of the three groups on day 1 and day 7 after surgery. **(B)** In the Sham, SAE model, and FMT-challenged SAE rats, Morris water maze was employed to monitor the moving track of rats in the pool. **(C)** The mobile distance and time percent during platform were recorded and calculated. *P < 0.05. **P < 0.01.

Intestinal flora homeostasis is closely related to cerebral diseases (Yamashiro, 2017). In the present study, cecal ligation and puncture (CLP)-induced SAE rats appeared to have a robustly decreased neurologic deficit score at day 1 postoperatively compared to the sham-operated rats that became no difference between them at day 7 postoperatively (**Figure 1A**). Once performing FMT, neural behavioral score was moderately restored in SAE rats, which did not affect their behavior on day 7 after surgery (**Figure 1A**). Based on the Morris water maze that was performed on day 9 after surgery, both the mobile distance and time percent during platform quadrant significantly reduced in SAE rats, while these were reversed in FMT-challenged SAE rats (**Figures 1B, C**). Additionally, in the SAE group, intestinal predominant flora including *Staphylococcus*, *Bifidobacterium*, *Lactobacillus*, *Clostridium*, and *Bacteroides* sharply declined, which were abrogated in the FMT-treated group (**Figures S1A, B**). The data prove that intestinal flora reconstruction will repair memory impairment in SAE rats.

### Fecal Microbiota Transplantation Relieves the Impaired Hippocampus

**Figure 2:**
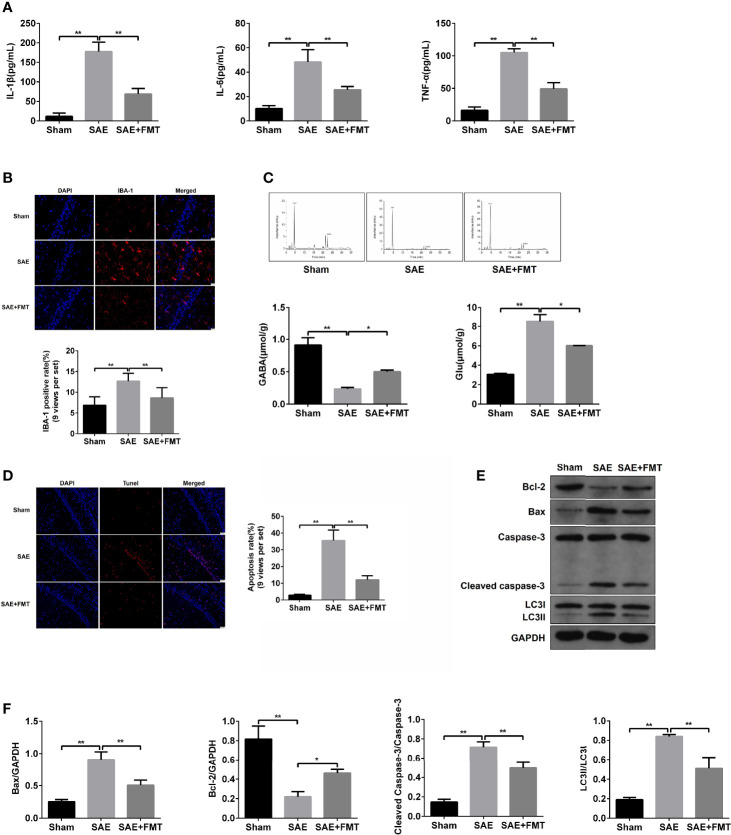
Effects of FMT on hippocampus impairment in SAE rats. **(A)** In hippocampus tissues of Sham-operated, SAE, and FMT-challenged SAE rats, the expression of inflammation cytokines interleukin (IL)-1β, IL-6, and TNF-α as determined by ELISA. **(B)** The expression of ionized calcium binding adaptor molecule (IBA) in paraffin-embedded hippocampus tissues as measured using IF staining. Red: IBA-1-positive signals; blue: DAPI. Relative quantitation of IBA-1-positive cells as shown below (nine views per set). **(C)** Based on the HPLC, the concentrations of glutamic acid (Glu) and gamma-aminobutyric acid (GABA) were detected in the hippocampus tissues of the three groups (Sham-operated, SAE, and FMT-challenged SAE rats). **(D)** TUNEL assay was used to determine the number of apoptotic cells in the hippocampus tissues obtained from Sham-operated, SAE, and FMT-challenged SAE rats. Relative quantitation of TUNEL-positive cells as shown in the right (nine views per set). **(E)** The protein levels of B-cell lymphoma (Bcl)-2, BCL2 associated X (BAX), cleaved caspase 3, and LC3II/I were detected by Western blotting assay in the hippocampus tissues of Sham, SAE, and FMT-exposed SAE rats. **(F)** Protein quantitative analysis of Bcl-1, BAX, cleaved caspase 3, and LC3II/I in panel **(E)** of this figure. *P < 0.05. **P < 0.01.

The impaired hippocampus will cause the neurobehavioral deficit. Subsequently, hippocampal tissues were harvested to assess the pathological lesions. In the results of H&E staining, morphological changes including karyopyknosis, cellular swelling, and irregular arrangement were observed in SAE rats compared to the Sham group. However, the hippocampal lesions were improved by the treatment with FMT (**Figure S2A**). The inflammation cytokines IL-1β, IL-6, and TNF-α in hippocampus were expressed highly in the model group and robustly reduced in FMT-exposed model rats (**Figure 2A**). The increased ionized calcium binding adaptor molecule (IBA)-1 expression distribution was observed in model rats and tended to normalize after FMT intervention (**Figures 2B**, **S2B**). Additionally, excitatory neurotransmitter glutamic acid (Glu) increased, while inhibitory neurotransmitter gamma-aminobutyric acid (GABA) decreased in SAE rats, which suggested that the hippocampus was impaired, and both were reversed by FMT (**Figure 2C**). Next, cell apoptosis was also evaluated in hippocampal tissues obtained from the three groups. More apoptotic cells (TUNEL-positive cells) and higher protein levels of cleaved-caspase 3 and Bax and lower Bcl-2 protein expression were verified in the hippocampus of SAE model rats and which effects were neutralized in model rats that received FMT (**Figures 2D**–**F**, **S2C**). Besides, FMT administration also abolished the elevated ratio of LC3II/I in SAE rats (**Figures 2E, F**). Possibly, FMT-mediated improvement on injured hippocampus is implicated in autophagic events.

### Fecal Microbiota Transplantation Prevents M1 Polarization of Mesenteric Lymph Nodes in Sepsis-Associated Encephalopathy Rats

**Figure 3:**
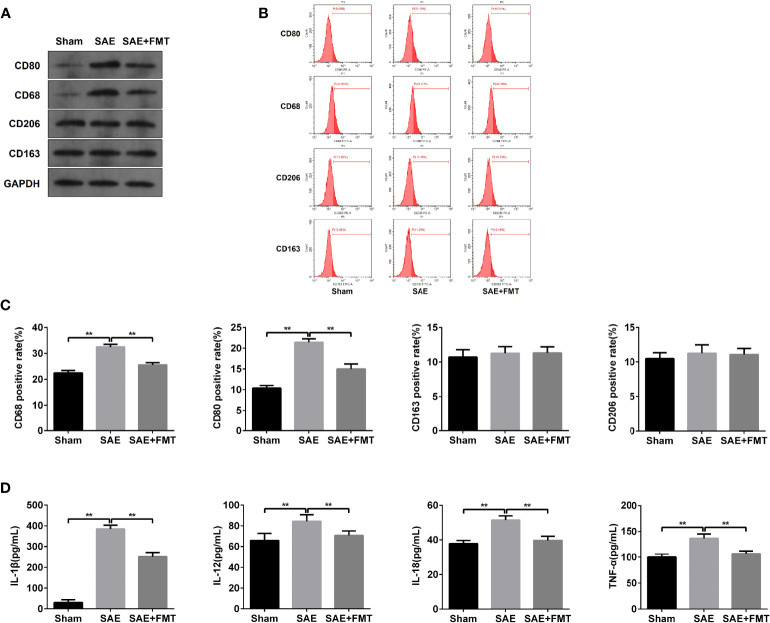
M1 macrophage polarization detection in mesenteric lymph nodes. **(A)** Mesenteric lymph nodes were harvested from the Sham-operated, SAE, and FMT-challenged SAE rats. Protein expression of CD80, CD68, CD206, and CD163 as determined by WB in the mesenteric lymph nodes from different rats. **(B)** CD80-, CD68-, CD206-, and CD163-positive cells were measured by flow cytometry. **(C)** Quantitative analysis of the proportion of CD80-, CD68-, CD206-, and CD163-positive cells. **(D)** The levels of IL-1β, IL-12, IL-18, and TNF-α were detected by ELISA kits. **P < 0.01.

The alterations of mucosal immune system always appear during sepsis-related disease (Wang et al., 2019). As shown in **Figure 3A**, the elevated protein levels of CD80 and CD68 and the proportion of CD80- and CD68-positive cells were confirmed in MLNs obtained from SAE rats compared to the Sham-operated rats (**Figures 3A**–**C**). By contrast, CD80/CD68 protein levels and CD80-/CD68-positive cell population reduced notably in MLNs of FMT-exposed model rats compared with the model group (**Figures 3A**–**C**). However, there were no changes of CD206/CD163 protein and CD206/CD163-positive cell number in MLNs in both SAE model and FMT-treated model groups in comparison to the Sham group (**Figures 3A**–**C**). CD80 and CD68 are the positive biomarkers of M1 macrophages, and CD206/CD163 were identified as the positive markers of M2 macrophages. Thus, FMT might inverse the M1 polarization of MLNs in SAE rats. To further prove the hypothesis, the cytokines secreted by M1 and M2 macrophages were determined. The levels of IL-1β, IL-12, IL-18, and TNF-α that were mainly produced by M1 macrophages decreased in MLNs of model group, and FMT administration prevented their secretion activity (**Figure 3D**). By contrast, there were no alterations in the secretion of CSF-1, IL-4, IL-10, TGF-β, and IL-13 in the three groups (**Figure S3A**). Additionally, the increased M1 macrophage-produced cytokines were observed in serum obtained from model rats and were abolished in FMT-treated model rats (**Figure S3B**). Consistently, serum M2 macrophage-related cytokines were not affected by SAE or FMT (**Figure S3C**). Collectively, FMT prevents M1 polarization of MLNs in SAE rats but does not affect M2 polarization.

### Intestinal Epithelial Cell-Derived Exosome of Sepsis-Associated Encephalopathy Rats Induces M1 Polarization

**Figure 4:**
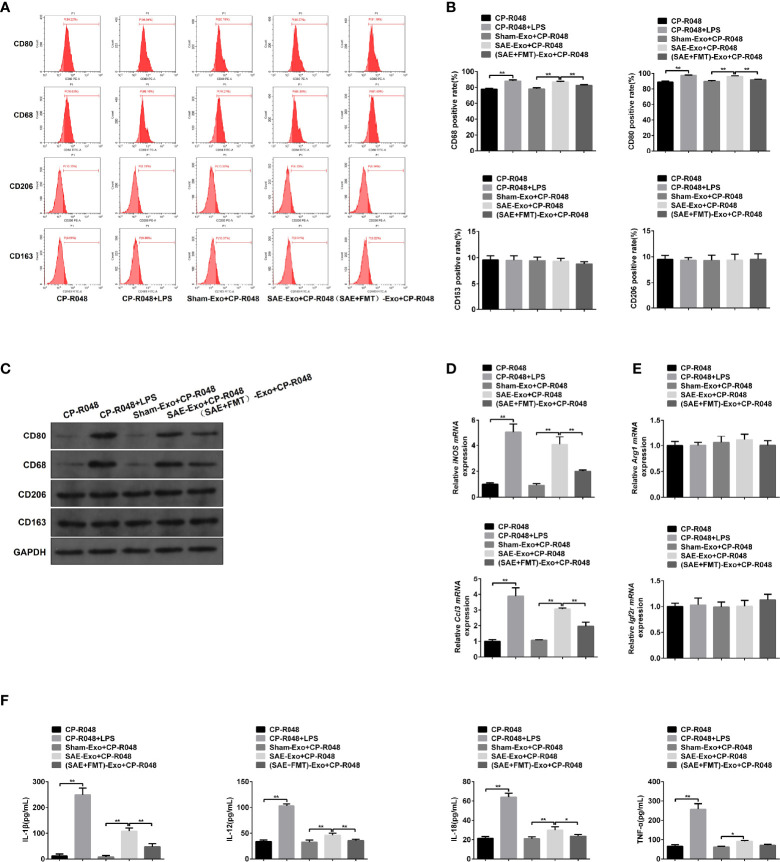
The association between M1 polarization and intestinal epithelial cell exosome. **(A)** CP-R048 cells were stimulated with LPS, or intestinal epithelial cell exosomes obtained from Sham-operated, SAE, and FMT-challenged SAE rats, respectively. Then, CD80-, CD68-, CD206-, and CD163-positive cells were measured by flow cytometry. **(B)** Quantitative analysis of the proportion of CD80-, CD68-, CD206-, and CD163-positive cells in panel **(A)** of this figure. **(C)** Protein expression of CD80, CD68, CD206, and CD163 as determined by WB in the CP-R048 cells stimulated with different reagents. Relative mRNA expression of inducible nitric oxide synthase (iNOS) and chemokine (C-C motif) ligand 3 (Ccl3) **(D)** and arginase (Arg1) and insulin-like growth factor 2 receptor (Igf2r) genes **(E)** in the CP-R048 cells treated with LPS and a different source of exosomes. **(F)** The levels of IL-1β, IL-12, IL-18, and TNF-α were detected by ELISA kits. *P < 0.05. **P < 0.01.

Next, the reason for M1 polarization in MLNs was assessed. As shown in **Figure S4A**, the exosomes secreted by IECs were isolated, and no morphological difference was found in the three groups (**Figure S4A**). The NTA results showed that particle diameter ranged from 30 to 100 nm. And a larger number of particles was observed in SAE group than the Sham group, which was moderately reduced in FMT-challenged model rats (**Figure S4B**). The isolated exosomes were also authenticated by WB assay using CD63, CD9, and CD81 antibodies and also confirmed its source using EpCAM antibody that was the positive marker of IEC (**Figure S4C**). Once CP-R048 macrophages were treated with the exosomes produced by rats of the three groups, the elevated CD80- and CD68-positive cell population and CD80/CD68 protein levels were found in the group of SAE exosome exposure, as well as the stimulation of positive drug LPS (**Figures 4A**–**C**). These changes in CP-R048 macrophages were counteracted by the stimulation of exosomes secreted from FMT-treated SAE rats (**Figures 4A**–**C**). However, the exosomes produced by the three types of rats did not modify the proportion of CD206-/CD163-positive cells and protein levels of CD206/CD163 (**Figures 4A**–**C**). Additionally, exosomes from SAE rats significantly induced the mRNA expression of inducible nitric oxide synthase (iNOS) and chemokine (C-C motif) ligand 3 (Ccl3) (**Figure 4D**) and the secretion of IL-1β, IL-12, IL-18, and TNF-α (**Figure 4F**), which were also found in LPS-exposed CP-R048 cells. By contrast, IEC-derived exosomes of FMT-treated model rats appeared to decline iNOS and Ccl3 mRNA levels and protein levels of IL-1β, IL-12, IL-18, and TNF-α compared to SAE exosome-treated CP-R048 cells (**Figures 4D, F**). However, exosomes from the three types of rats did not regulate the expression of Arg1 and Igf2r and the production of CSF-1, IL-4, IL-10, TGF-β, and IL-13, which were all M2 macrophage-related molecules (**Figures 4E**, **S5**). Therefore, IEC exosome from SAE rats can induce *ex vivo* M1 polarization.

### Inhibition of Exosome Secretion Represses M1 Polarization of Mesenteric Lymph Nodes in Sepsis-Associated Encephalopathy Rats

**Figure 5:**
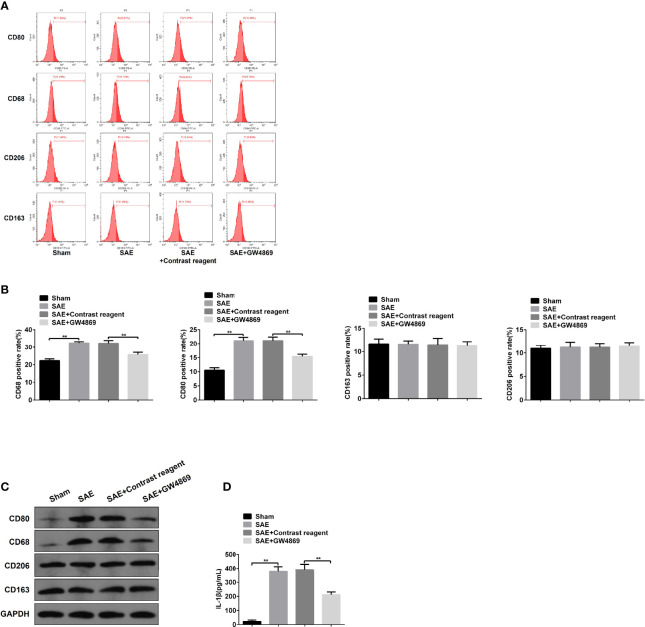
Effects of GW4869 on M1 polarization in SAE rats. **(A)** Rats were processed with Sham control surgery and CLP treatment. In CLP-induced SAE rats, rats were challenged with control reagent or GW4869 administration. And CD80-, CD68-, CD206-, and CD163-positive cells in mesenteric lymph nodes were measured by flow cytometry. **(B)** Quantitative analysis of the proportion of CD80-, CD68-, CD206-, and CD163-positive cells in panel **(A)** of this figure. **(C)** Protein expression of CD80, CD68, CD206, and CD163 as determined by WB in the isolated cells from different rats. **(D)** The level of IL-1β was detected by ELISA kits. **P < 0.01.

To investigate the role of intestinal exosomes on polarization of macrophages, SAE rats were challenged by intraperitoneal injection of exosome secretion inhibitor GW4869. Firstly, the exosomes from Sham-operated, SAE, and SAE treated with control reagent and GW4869 were successfully isolated and authenticated by TEM, NTA, and WB assays (**Figures S6A–C**). GW4869 administration significantly reduced the elevated exosome concentration of SAE rats (**Figure S6B**). In the MLNs, the increased CD80-/CD68-positive cell population and protein levels of CD80/CD68 in SAE rats were abolished in GW4869-challenged SAE rats (**Figures 5A**–**C**). In addition to that, the production of IL-1β in MLNs of SAE rats significantly elevated, which was sharply inhibited by the administration of GW4869 (**Figure 5D**). As expected, GW4869 exposure also could not regulate the proportion of CD206/CD163-positive cells (**Figures 5A**–**C**). Thus, M1 polarization of MLNs in SAE rats depends on the secretion of intestinal exosomes.

### IL-1β Is Required for Hippocampus Impairment in Sepsis-Associated Encephalopathy Rats

**Figure 6:**
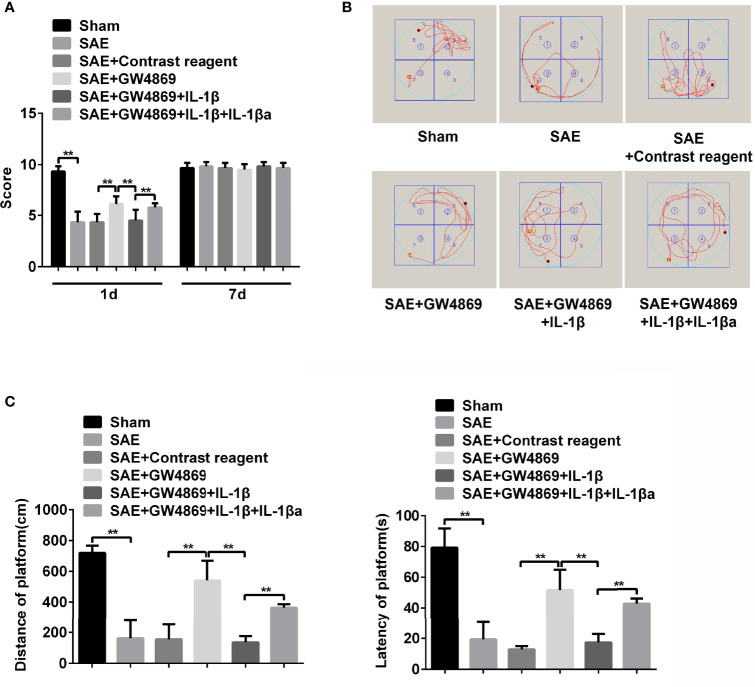
Role of IL-1β in memory impairment of SAE rats. **(A)** Rats were processed with Sham control surgery and CLP treatment. Then, the SAE rats were divided into five groups: Sham, SAE, SAE treated with control reagent, SAE treated with IL-1β, SAE treated with IL-1β and IL-1β antagonist. Then, neurological behavior score was measured. **(B)** Morris water maze was used to monitor the moving track of rats in the pool. **(C)** The distance and time percent on platform were recorded and calculated. **P < 0.01.

Subsequently, the effect of GW4869 on memory impairment in SAE rats was also verified. As shown in **Figure 6**, the declined neural behavioral score of SAE rats was observably restored by the treatment with GW4869 (**Figure 6A**). Additionally, the decreased displacement distance and latency time in platform of Morris water maze were robustly reversed by GW4869 administration (**Figures 6B, C**). The data indicated that inhibition of exosome secretion facilitated the improvement of memory impairment in SAE rats. Interestingly, once recombinant IL-1β was delivered to the left hippocampus, the GW4869-exerted obvious ameliorative effects on memory impairment including behavioral score, distance, and latency time of platform were all abolished (**Figures 6A**–**C**). However, exogenous IL-1β-mediated reappeared disease symptoms in GW4869-exposed SAE rats were also neutralized by antagonist of IL-1β (**Figures 6A**–**C**). The expression of IL-1β in different groups was shown in **Figure S7**. GW4869 administration notably reduced the levels of serum and hippocampal IL-1β compared to that of SAE rats. The addition of IL-1β antagonist effectively suppressed serum and hippocampal IL-1β levels (**Figures S7A, B**). Thus, IL-1β acts as a key switch during memory impairment in SAE rats.

**Figure 7:**
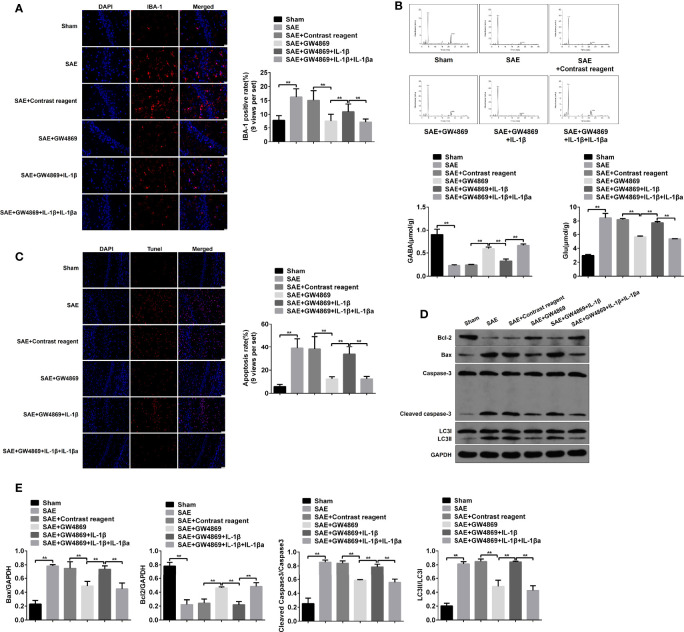
Role of IL-1β in hippocampus impairment of SAE rats. **(A)** In hippocampus tissues of Sham-operated, SAE, SAE treated with control reagent, SAE treated with IL-1β, and SAE treated with IL-1β and IL-1β antagonist rats, the expression of IBA-1 in paraffin-embedded hippocampus tissues as measured using IF staining. Relative quantitation of IBA-1-positive cells as shown in the right (nine views per set). **(B)** The concentrations of Glu and GABA were detected by HPLC in the hippocampus tissues. **(C)** TUNEL assay was employed to determine the apoptotic cells in the hippocampus tissues. Relative quantitation of TUNEL-positive cells as shown in the right (nine views per set). **(D)** The protein levels of Bcl-1, BAX, cleaved caspase 3, and LC3II/I were detected by WB in the hippocampus tissues. **(E)** Protein quantitative analysis of Bcl-1, BAX, cleaved caspase 3, and LC3II/I in panel **(D)** of this figure. **P < 0.01.

Additionally, the pathological changes of hippocampus were further determined. As expected, pathological damages in model rats were ameliorated by the addition of GW4869, while simultaneous injections with GW4869 and recombinant IL-1β abrogated the improvement effect of GW4869. By contrast, reinhibition of IL-1β restored the impaired hippocampus (**Figure S7C**). Consistently, the elevated IBA-1 expression and Glu secretion and the decreased GABA level in SAE rats were inversed by GW4869 treatment, which were reinduced by exogenous IL-1β and relimited by IL-1β antagonist (**Figures 7A, B**). Moreover, the increased apoptotic cells (TUNEL-positive cells) and protein levels of cleaved caspase 3 and Bax and the reduced Bcl-2 protein level in hippocampus of model rats were significantly reversed in GW4869-treated model rats. However, the administration of recombinant IL-1β abolished the effects of GW4869, and IL-1β inhibition further appeared to be a restorative effect of GW4869 (**Figures 7C**–**E**). As mentioned above, cell autophagy was activated along with the increased LC3II/I ration in hippocampus of SAE rats. GW4869 exposure inactivated but exogenous IL-1β reactivated autophagy activity of hippocampus (**Figures 7D, E**). Therefore, exosome-mediated activation of autophagy may be implicated in hippocampus impairment of SAE rats.

### Autophagy Mediates Intestinal Epithelial Cell Exosome-Induced Damage and Apoptosis of Hippocampal Neurons

**Figure 8:**
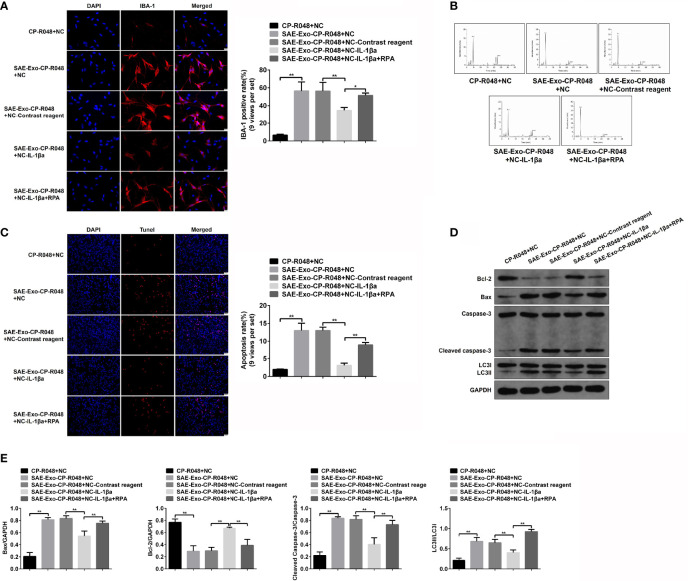
Effects of autophagy in IL-1β-induced neuron injury. **(A)** Firstly, CO-R048 cells were treated with intestinal epithelial cell exosome from Sham and SAE rats. The supernatants were collected for the incubation of H19-7 cells. H19-7 cells were also exposed to control reagent, IL-1β antagonist, and autophagy activator rapamycin. Thus, there were five groups of cells: H19-7 cells treated with control supernatant, H19-7 cells treated with disease supernatant, control reagent-exposed H19-7 cells treated with control supernatant, IL-1β antagonist-exposed H19-7 cells treated with control supernatant and IL-1β antagonist, and IL-1β antagonist and rapamycin-exposed H19-7 cells treated with disease supernatant. Relative quantitation of IBA-1-positive cells as shown in the right (nine views per set). **(B)** In the above cells, the concentrations of Glu and GABA were detected by HPLC. **(C)** Apoptotic cells were determined by TUNEL assay in these cells. Relative quantitation of TUNEL-positive cells as shown in the right (nine views per set). **(D)** The protein levels of Bcl-1, BAX, cleaved caspase 3, and LC3II/I were detected by WB in the cells. **(E)** Protein quantitative analysis of Bcl-1, BAX, cleaved caspase 3, and LC3II/I in panel **(D)** of this figure. *P < 0.05. **P < 0.01.

To explore the direct role and regulatory mechanism of intestinal IL-1β on hippocampal neurons, the supernatant of SAE exosome-treated CP-R048 cells was collected to incubate with neurons. As shown in **Figure S8A**, the elevated IL-1β expression was observed in SAE exosome-exposed CP-R048 cells compared to the Sham exosome-exposed cells (**Figure S8A**). Once hippocampal neurons H19-7 were incubated with the supernatant of SAE exosome-challenged CP-R048 cells, the elevation of IBA-1 level and Glu production and the decrease of GABA were found compared to supernatant from normal cells. However, inhibition of IL-1β using its antagonist in H19-7 significantly prevented the role of supernatant from SAE exosome-challenged CP-R048 cells (**Figures 8A, B**). As mentioned above, IL-1β antagonist reduced the ratio of LC3II/I, implying autophagy was involved in the cellular process. Here, the rapamycin (RPA)-induced reactivation of autophagy in H19-7 cells robustly abolished the ameliorative role of IL-1β antagonist (**Figures 8A, B**). Of note, SAE exosome-exposed CP-R048 supernatant mediated the elevation of apoptotic cells (TUNEL-positive cells), Bax and cleaved caspase 3 protein expression, and the decrease of Bcl-2 protein level, which were all reversed by the inhibition of IL-1β in H19-7 cells. The apoptotic events also were relaunched by RPA-mediated autophagy activation (**Figures 8C**–**E**). Based on the ratio of LC3II/I, disease supernatant-induced autophagy flux and RPA-mediated reactivation of autophagy were verified in H19-7 cells. Therefore, intestinal IL-1β can cause damage and apoptosis of hippocampal neurons in an autophagy-dependent manner.

## Discussion

During the last years, progress has been made in our understanding of the pathophysiology of sepsis; however, no target treatment for SAE is available (Molnar et al., 2018). Besides, it is acknowledged that inflammatory cytokines are key players in mediating brain injury and behavioral cognitive impairment (Briones et al., 2013; Newell et al., 2018). Hyperinflammatory response is thought to be the basic feature of systemic infection, and in septic shock patients, the levels of pro-inflammatory factors such as TNF-α, IL-1β, and IL-6 are elevated, which are closely related to the clinical severity and prognosis of SAE patients (Xiao et al., 2011). Thus, inflammation modification is a good choice for SAE therapy. In the present study, the occurrence of SAE is accompanied by cognitive impairment and intestinal flora disorders, which is attributed to M1 polarization in MLNs caused by IEC-derived exosomes. Furthermore, it is demonstrated that SAE-induced cognitive impairment is dependent on IL-1β. Possibly, the data may provide a novel treatment strategy for SAE *via* targeting gut–brain signal axis.

The gut microbiota participates in the occurrence of many brain diseases including Parkinson’s disease, neurodegenerative diseases, SAE, and Alzheimer’s disease (Mulak and Bonaz, 2015; Quigley, 2017; Yang et al., 2018), and FMT has been applied in the treatment of several brain diseases including SAE (Cui et al., 2016; Li et al., 2017; Zhang et al., 2018; Fang, 2019). In our result, we observed that SAE rats suffered cognitive impairment, hippocampal injury, inflammation, and autophagy activation, which can be relieved by FMT treatment. It is indicated that FMT-mediated improvement of SAE is related to the reduction of cell inflammation and the inhibition of autophagy in the hippocampus. SAE is a diffuse brain dysfunction caused by an unbalanced inflammatory response, which is proven to be closely related to the imbalance of intestinal microbiota (Sasmita, 2019). It is reported that MLNs are involved in intestinal inflammation (Kawabe et al., 2016). Besides, the number of infiltrating inflammatory cells, cytokines, and CD169+ macrophages increases significantly in MLNs of mouse colitis model (Li et al., 2017). Intestinal macrophage exhibits immune response depending on the gut microbiota, and intestinal flora can affect macrophage polarization (Schulthess et al., 2019). Therefore, macrophage polarization and the following inflammation accumulation or cytokine production may be the bridge linking intestinal microbiota disorder and encephalopathy. Here, M1 macrophages were activated in the MLN, and inflammation factors were accumulated in MLN and circulatory system in SAE rats, which were significantly relieved after FMT treatment. These results indicate that the accumulation of inflammatory factors caused by M1 polarization may enter the circulatory system and interfere with the progress of SAE.

Location of IECs harbors a strategic position between the external environment and the most extended lymphoid tissue in the body (Mallegol et al., 2005). A number of studies have described the ability of IECs to secrete immunologically active extracellular vesicles (EVs), including exosomes, which could deliver important molecules such as proteins, DNA, and several inflammatory cytokines, thereby affecting the immune environment (van Niel and Heyman, 2002; Xu et al., 2016). Intestinal epithelium-derived luminally releases extracellular vesicles in sepsis to suppress cytokine release in mucosal inflammation (Appiah et al., 2020). In our study, exosomes derived from IECs induced M1 polarization with increasing secretion of IL-1β in SAE rats, which could be inhibited by exosome inhibitor GW4869. Besides, FMT treatment inhibited M1 polarization in the MLN area depending on IEC exosomes. The finding suggests that IECs can regulate the immune response of MLNs through exosome release, thereby affecting IL-1β production. However, bioactive molecules carried by exosomes inducing the immune response need further study.

Cytokine secretion mediates brain dysfunction, and the increases of IL-1β, IL-6, and TNF-α are involved in cognitive impairment after sepsis (Imamura et al., 2011; Mina et al., 2014). During sepsis, the release of pro-inflammatory molecule IL-1β leads to systemic inflammation, which subsequently affects the gut integrity (Van Looveren et al., 2020). Besides, in inflammatory bowel disease, levels of IL-1β in MLNs increase significantly, and inhibition of NLRP3/IL-1β signal axis is thought to improve the symptoms of SAE (Sui et al., 2016). Our results suggested that the cognitive impairment and hippocampal apoptosis of SAE rats depended on IL-1β. It is speculated that the IL-1β released by M1 polarization may flow to the hippocampus through the circulatory system, thereby affecting the hippocampus injury.

Autophagy is thought to play an essential role in sepsis (Oami et al., 2017), which represents the cytoprotective mechanism against microbial infection in the early stage of sepsis (Kemp, 2017). Impaired autophagy pathway may cause brain damage in SAE (Liu et al., 2017). Moreover, the autophagy process of hippocampal neurons in septic rats is relevant to NF-κB signaling pathway, which is responsible for inflammation occurrence (Zi et al., 2015). It is reported that autophagy in hippocampus nerve cells affects the progression of SAE (Huang et al., 2017). Our data showed that the autophagy was activated in the hippocampus of SAE rats, which could be relieved in FMT-SAE rats. It is indicated that FMT improves nerve damage possibly through the brain–gut axis by affecting the autophagy activity of neuronal cells in hippocampus. Additionally, the activation of autophagy limited the functions of IL-1β antagonists, further demonstrating that autophagy activation is required for IL-1β-dependent hippocampal tissue damage in SAE rats. Overall, these results may provide evidence for treating SAE through targeting gut–brain–autophagy signal axis clinically. However, we did not perform the other behavioral analyses in the SAE model mice and the pathological changes in other brain areas excluding hippocampus. In addition, whether other inflammatory factors secreted from macrophages were involved in these events remained unclear.

In summary, FMT treatment improves SAE symptoms, accompanied by the decreased M1 polarization in MLNs. IEC-derived exosomes induce M1 polarization that subsequently mediated the secretion of IL-1β in MLNs and in the peripheral circulation, resulting in cognitive impairment, inflammation, and hippocampal damage in SAE rats. Possibly, intestinal flora disorder promotes the release of IEC-derived exosomes that elevate the SAE progression *via* M1 polarization and IL-1β secretion in MLNs. Our findings reveal a novel regulatory mechanism underlying intestinal flora disorder-induced SAE.

## Data Availability Statement

The datasets presented in this study can be found in online repositories. The names of the repository/repositories and accession number(s) can be found below: https://www.ncbi.nlm.nih.gov/bioproject/PRJNA767832/.

## Ethics Statement

Animal experiments were carried out with approval by the Ethics Committee of the Affiliated Hangzhou First People’s Hospital and conducted in accordance with the China Code of Practice for the Care and Use of Animals for Scientific Purposes.

## Author Contributions

WH and YZ conceived the idea. WH drafted the article. SX and YW designed and performed the experiments, analyzed the data, and designed the figures. YW, CW and WP contributed to Western blotting assay and qRT assay. All authors discussed the results and edited this article. All authors contributed to the article and approved the submitted version.

## Funding

This work was supported by the Zhejiang Provincial Natural Science Foundation of China (Grant No. LY19H030007), the Zhejiang Provincial Medical and Health Technology Project (Grant No. 2022KY252), and the Construction Fund of Medical Key Disciplines of Hangzhou (2020–2024).

## Conflict of Interest

The authors declare that the research was conducted in the absence of any commercial or financial relationships that could be construed as a potential conflict of interest.

## Publisher’s Note

All claims expressed in this article are solely those of the authors and do not necessarily represent those of their affiliated organizations, or those of the publisher, the editors and the reviewers. Any product that may be evaluated in this article, or claim that may be made by its manufacturer, is not guaranteed or endorsed by the publisher.
