# Supplementary material for: Intestinal Epithelial Cell Exosome Launches IL-1β-Mediated Neuron Injury in Sepsis-Associated Encephalopathy
Source: Front Cell Infect Microbiol. 2022 Jan 17;11:783049. doi: 10.3389/fcimb.2021.783049 (PMC8801738; doi:10.3389/fcimb.2021.783049)
Supplement: Supplementary file 1 [file Table_1.docx]

Supplementary Material

# Supplementary Figures and Tables

## Supplementary Tables

**Table S1. Primer sequences used for RT-PCR**

| **Gene** | **Primer** | **Sequence** | **Size** |
| --- | --- | --- | --- |
| Rat GAPDH | Forward | 5‘- ACAGCAACAGGGTGGTGGAC-3’ | 253 bp |
|  | Reverse | 5‘- TTTGAGGGTGCAGCGAACTT-3’ |  |
| Rat iNOS | Forward | 5‘-AAGGGATCTTGGAGCGAGTT-3’ | 159 bp |
|  | Reverse | 5‘- GAGGGGTAGTGATGTCCAGG-3’ |  |
| Rat Arg1 | Forward | 5‘-GCCCATTCACCTGAGTTTTGA -3’ | 259 bp |
|  | Reverse | 5‘-ATTACCTTCCCGTTTCGTTCC -3’ |  |
| Rat Ccl3 | Forward | 5‘- CAGCCGGGTGTCATTTTCCT -3’ | 232 bp |
|  | Reverse | 5‘- GTGGCTACTTGGCAGCAAAC-3’ |  |
| Rat Igf2r | Forward | 5‘-GGCCTCTGTTCATCAGTGCT -3’ | 160 bp |
|  | Reverse | 5‘- CCAGCTTTGCCACTTAGGGA -3’ |  |

## Supplementary Figures

**
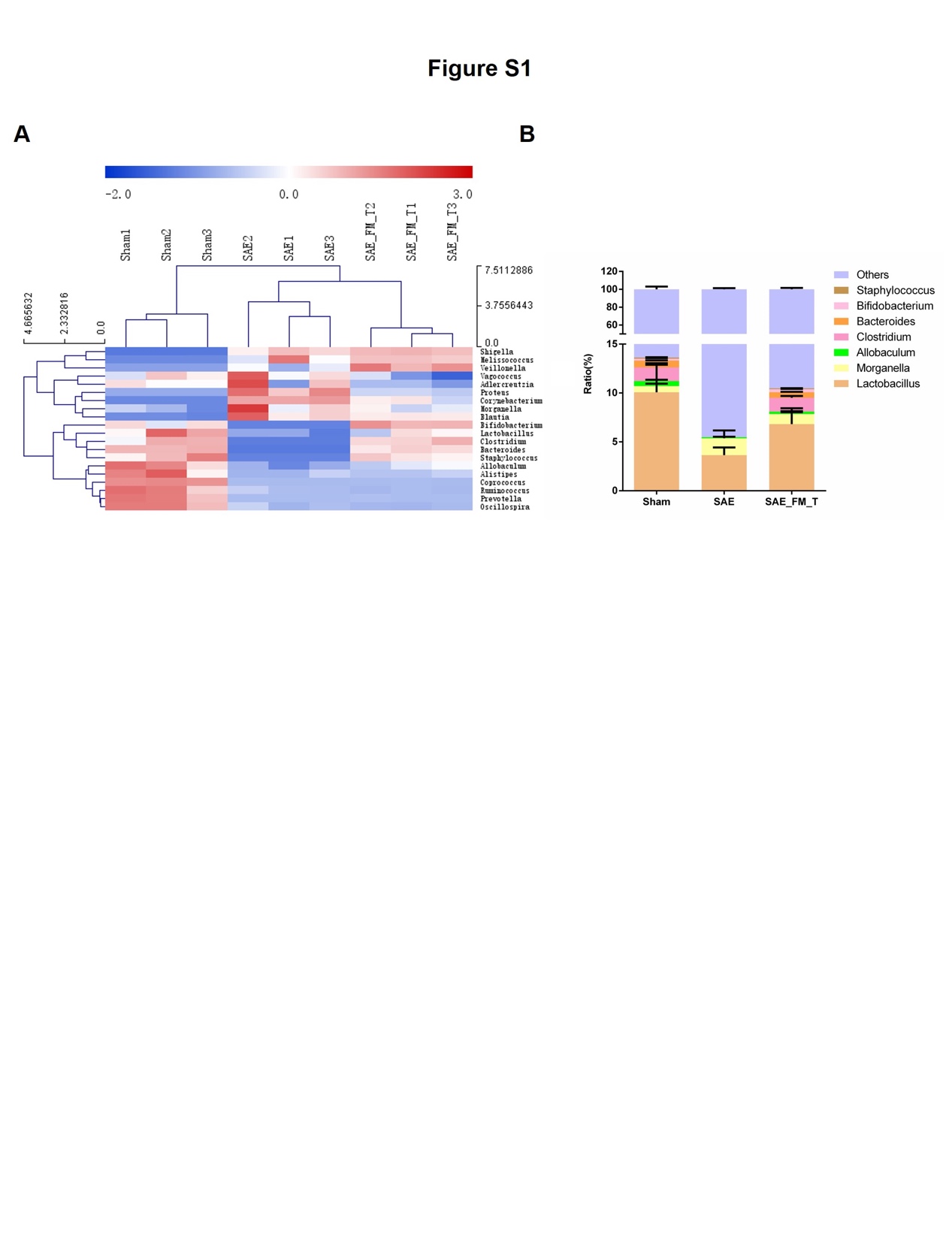
**

**Figure S1 16s rRNA sequencing. (A)** The rats were processed with Sham control and cecal ligation and puncture surgeries, respectively. The SAE rats were received with control reagent and FMT. After modeling, the fresh feces were collected and performed by 16s rRNA sequencing. The different intestinal flora was presented in the cluster diagram. **(B)** The different intestinal flora was presented in a histogram.


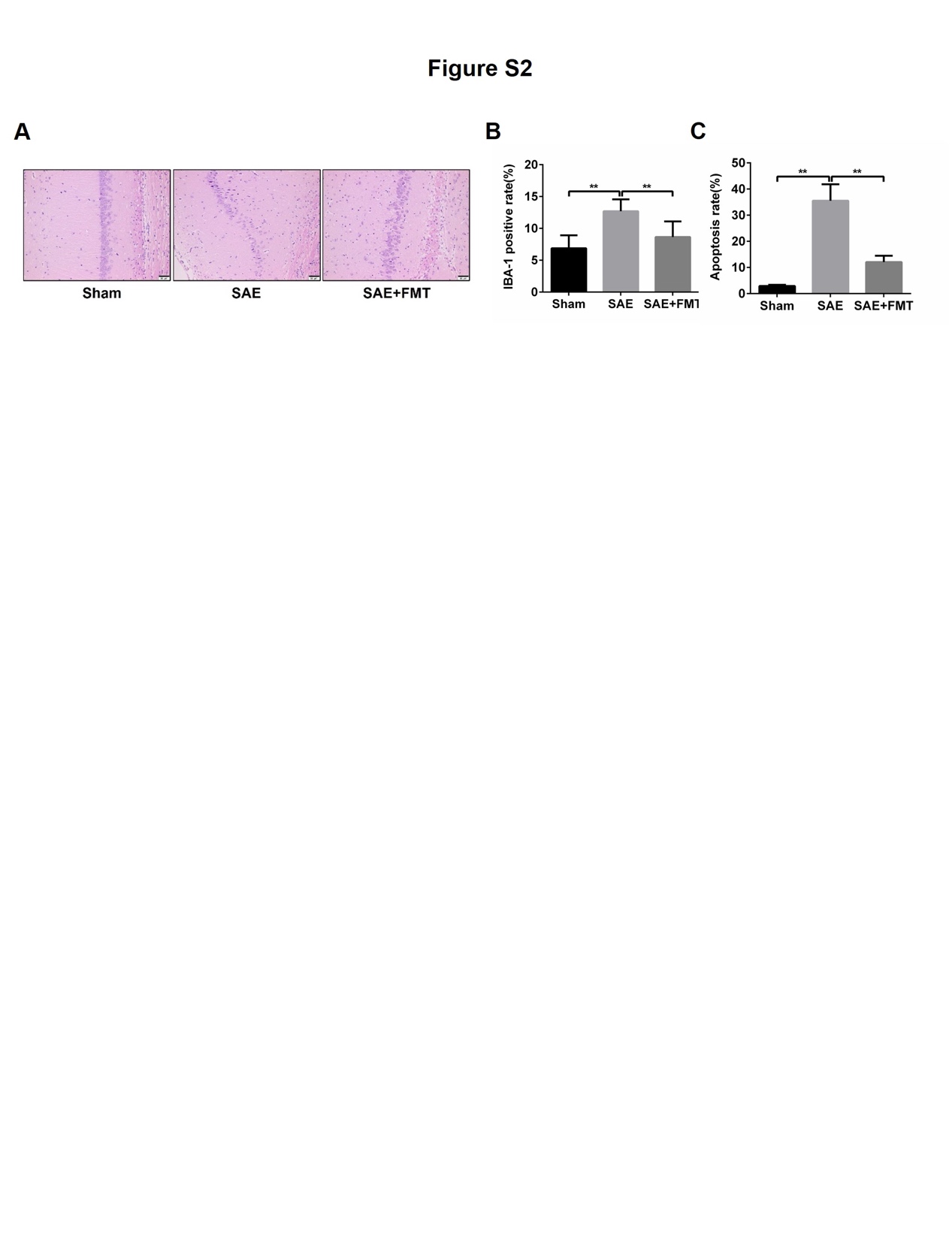


**Figure S2 Hippocampus impairment of SAE rats. (A)** Hematoxylin-eosin staining of hippocampus tissue in Sham, SAE and FMT-challenged SAE rats. **(B)** Quantitative analysis of IBA-1-positive cells in Sham, SAE and FMT-challenged SAE rats. **(C)** Quantitative analysis of the proportion of apoptotic cells in Sham, SAE and FMT-challenged SAE rats. **P<0.01.


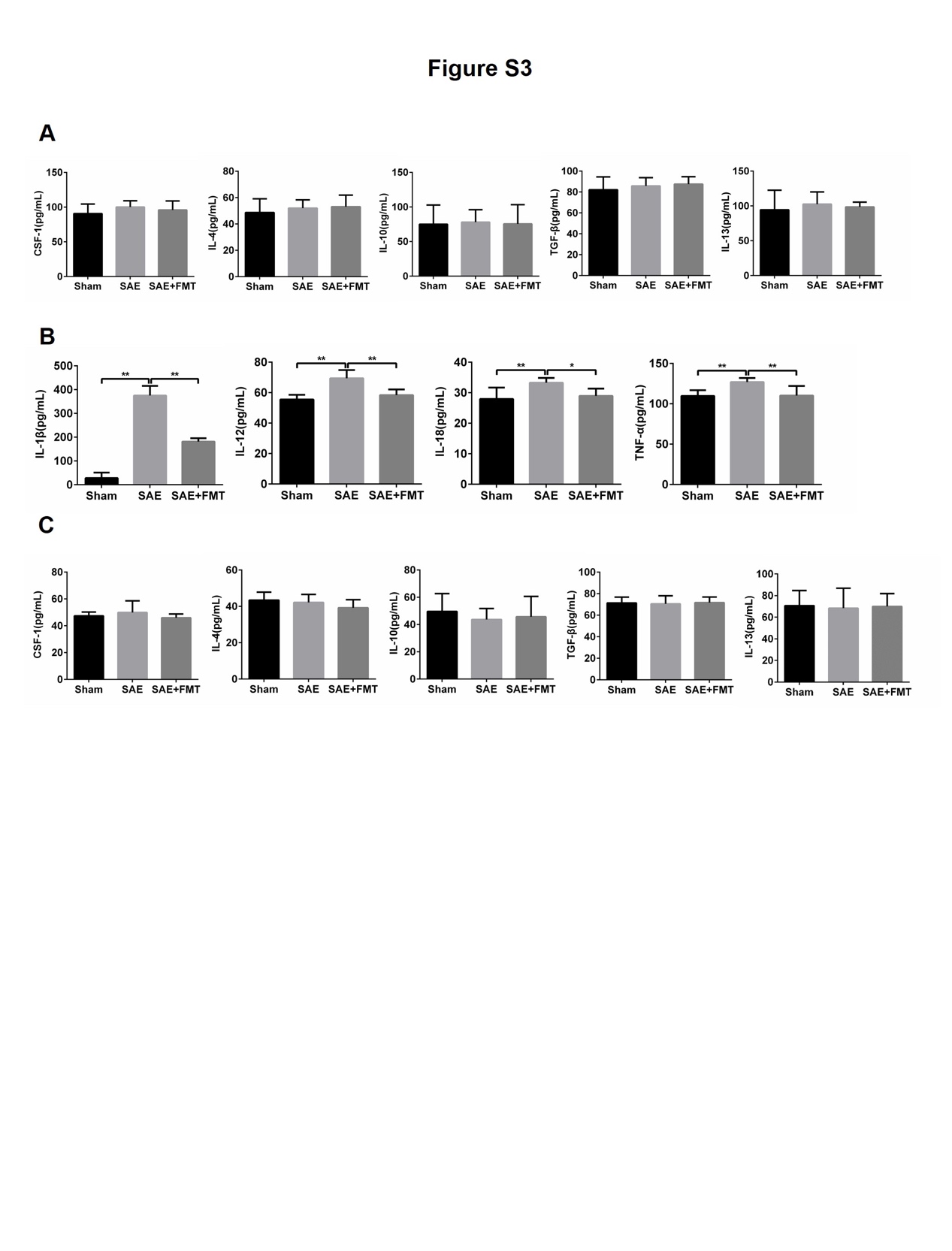


**Figure S3 Macrophage polarization detection. (A)** The secretion of CSF-1, IL-4, IL-10, TGF-β and IL-13 in hippocampus tissue as determined by ELISA kits. The levels of serum IL-1β, IL-12, IL-18 and TNF-α **(B)** and serum CSF-1, IL-4, IL-10, TGF-β and IL-13 **(C)** were detected by ELISA kits. *P<0.05. **P<0.01.


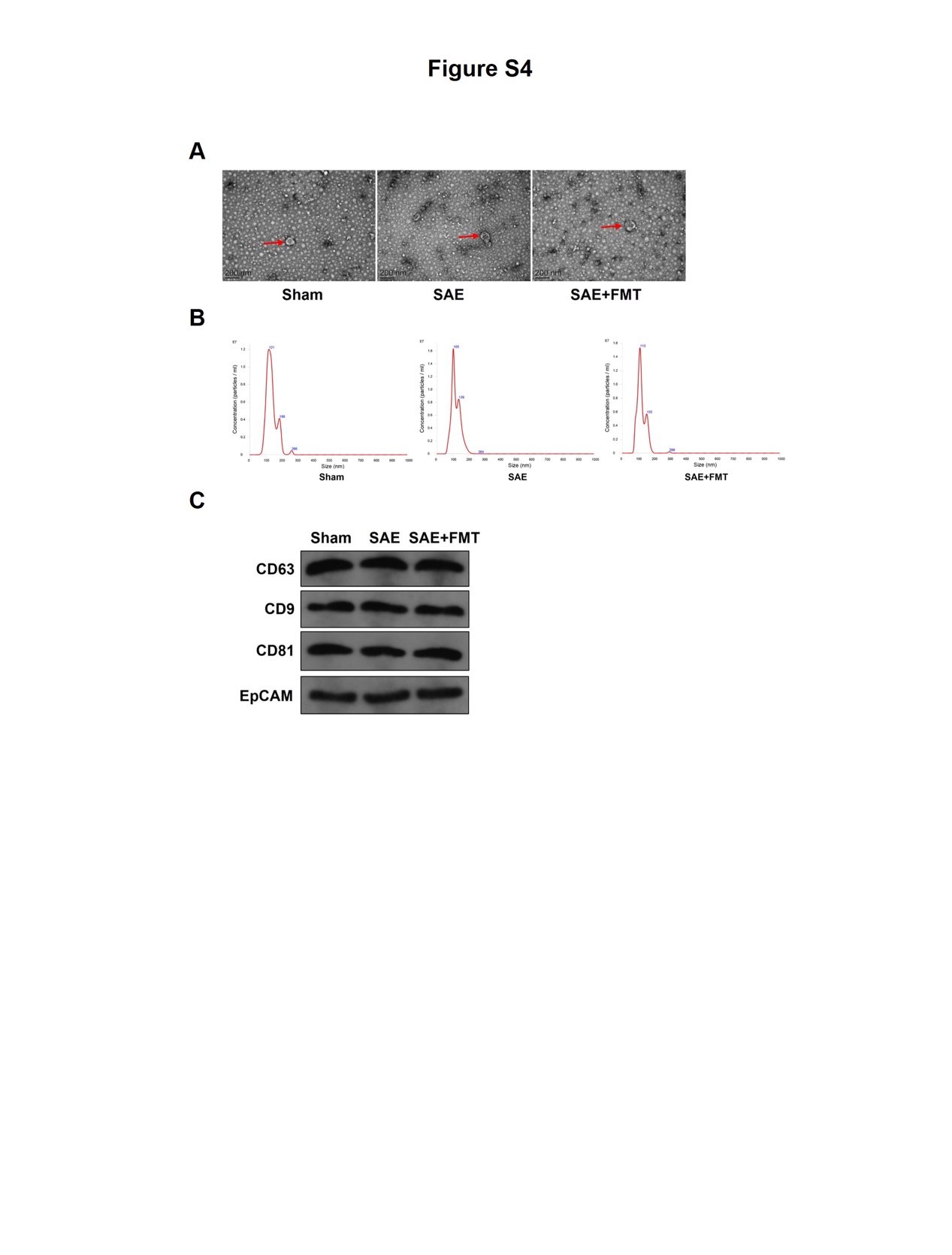


**Figure S4 Isolation and authentication of exosomes. (A)** Exosomes were isolated from mesenteric lymph nodes and identified by TEM. **(B)** The diameter and concentration of exosomes were measured by NTA. **(C)** Exosomes were further verified by WB using CD63, CD9, CD81 and EpCAM antibodies.


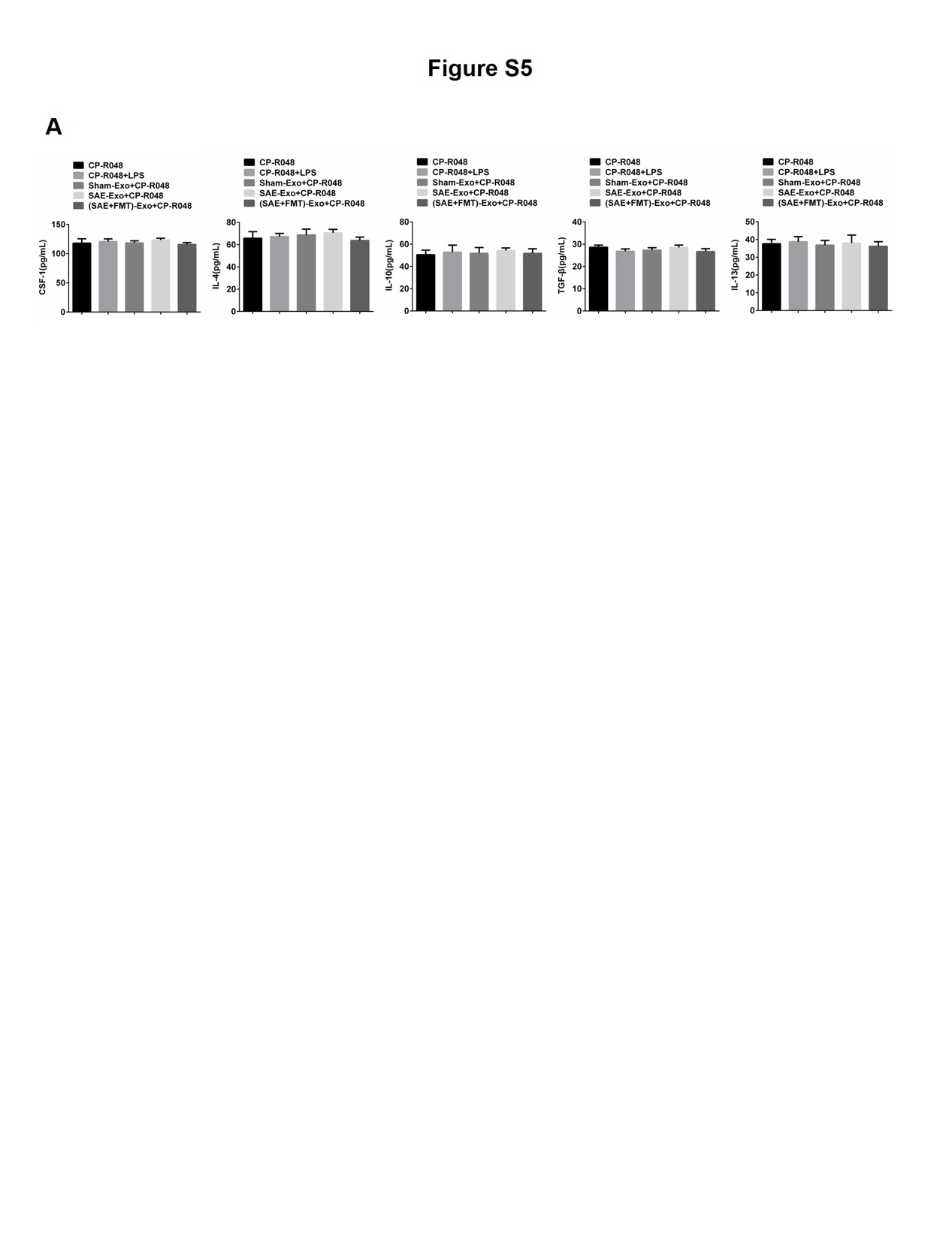


**Figure S5 Detection of M2 macrophage-related cytokines. (A)** CP-R048 cells were stimulated with LPS, intestinal epithelial cell exosomes obtained from Sham-operated rats, SAE and FMT-challenged SAE rats. The secretion of CSF-1, IL-4, IL-10, TGF-β and IL-13 in CP-R048 cells as determined by ELISA kits.

**
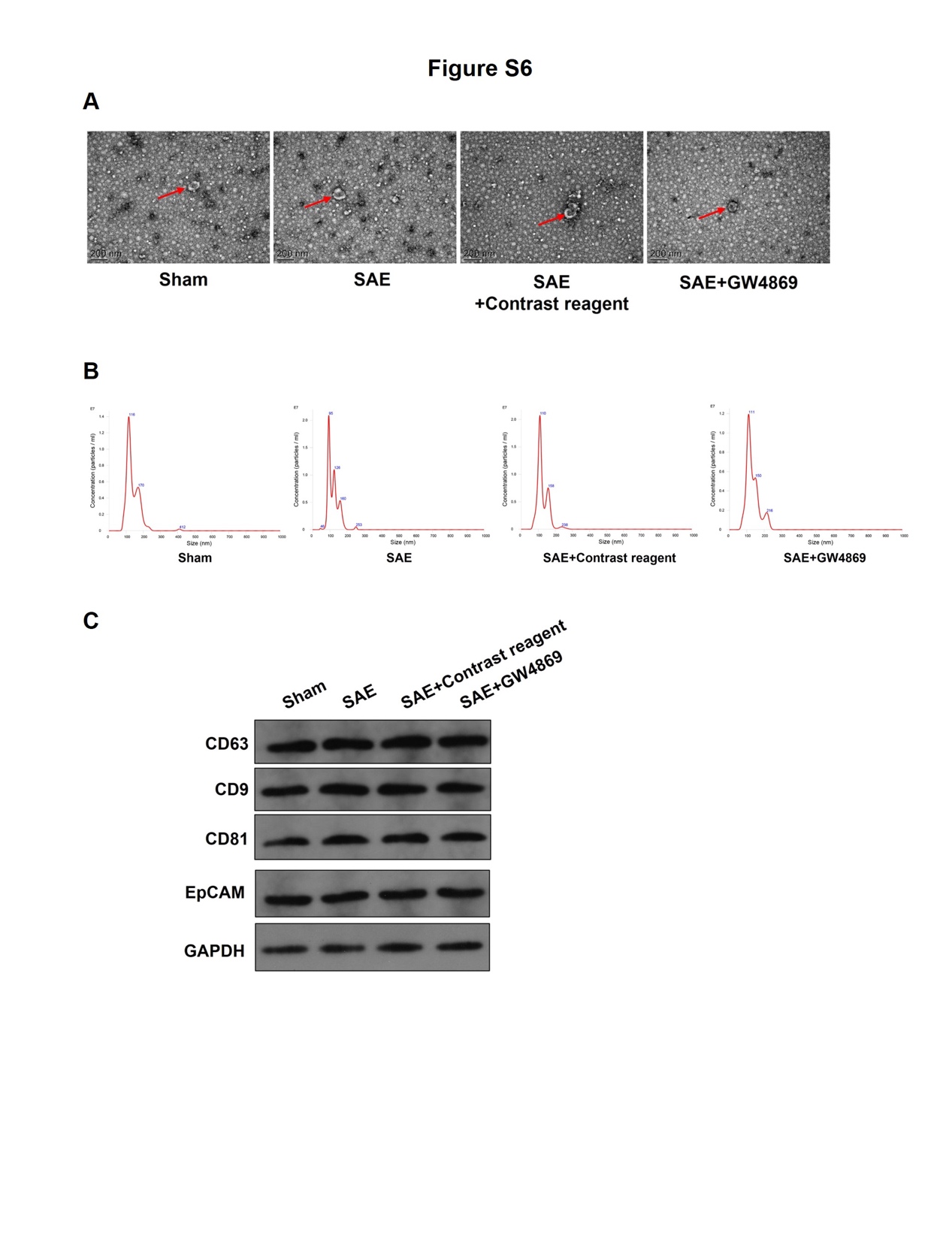
**

**Figure S6 Exosome measurement after GW4869 administration. (A)** Rats were processed with Sham control surgery and CLP treatment. In CLP-induced SAE rats, rats were challenged with control reagent or GW4869 administration. Then Exosomes were isolated from mesenteric lymph nodes and identified by TEM. **(B)** The diameter and concentration of exosomes were measured by NTA. **(C)** Exosomes were further verified by WB using CD63, CD9, CD81 and EpCAM antibodies.


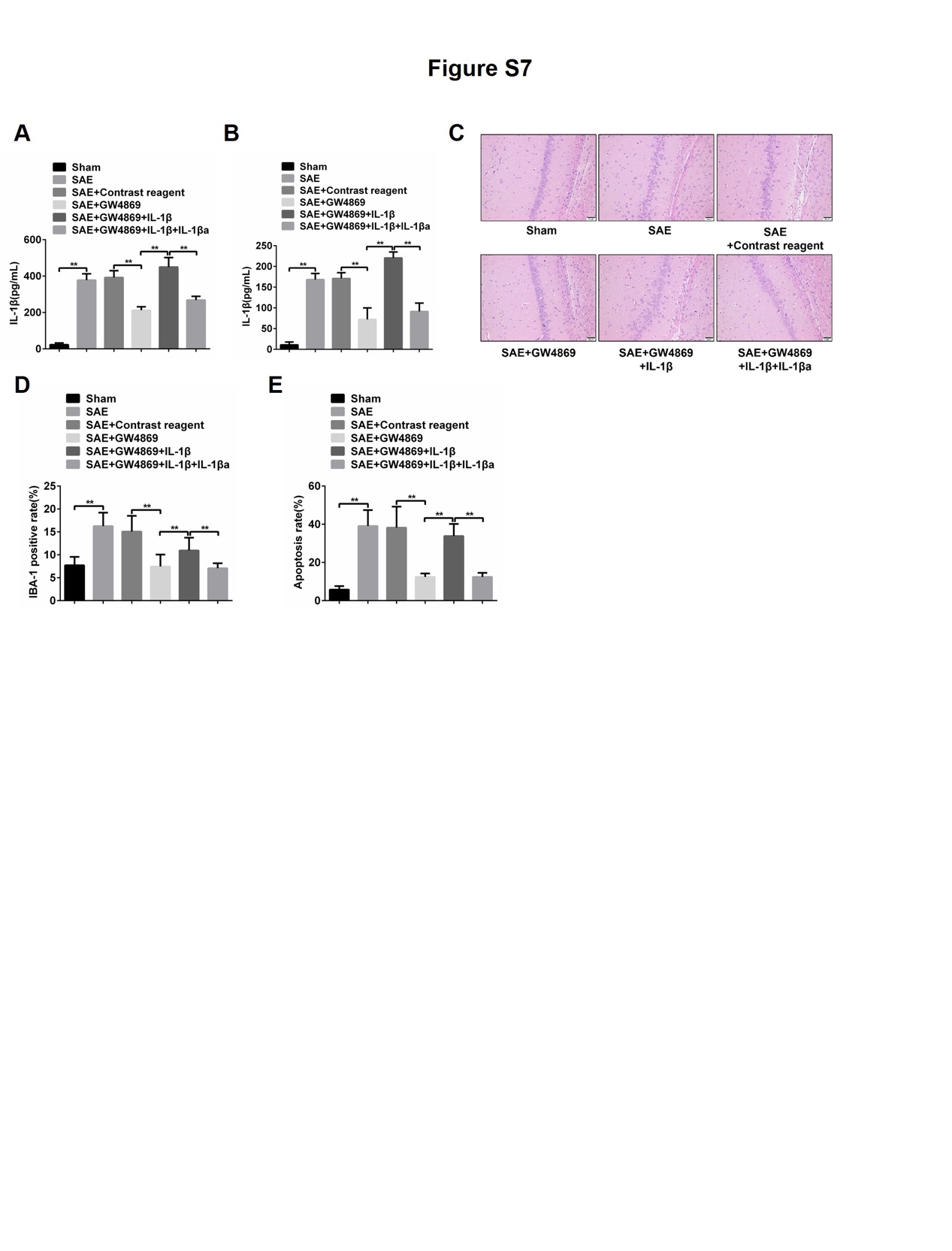


**Figure S7 Detection of hippocampus impairment in IL-1β-challenged SAE rats.** Rats were processed with Sham control surgery and CLP treatment. Then the SAE rats were divided into five groups: Sham, SAE, SAE treated with control reagent, SAE treated with IL-1β and SAE treated with IL-1β and IL-1β antagonist. Serum **(A)** and hippocampal **(B)** IL-1β were determined by ELISA kits. **(C)** Hematoxylin-eosin staining of hippocampus tissue. **(D)** Quantitative analysis of IBA-1-positive cells in these rats. **(E)** Quantitative analysis of the proportion of apoptotic cells in the above rats. **P<0.01.


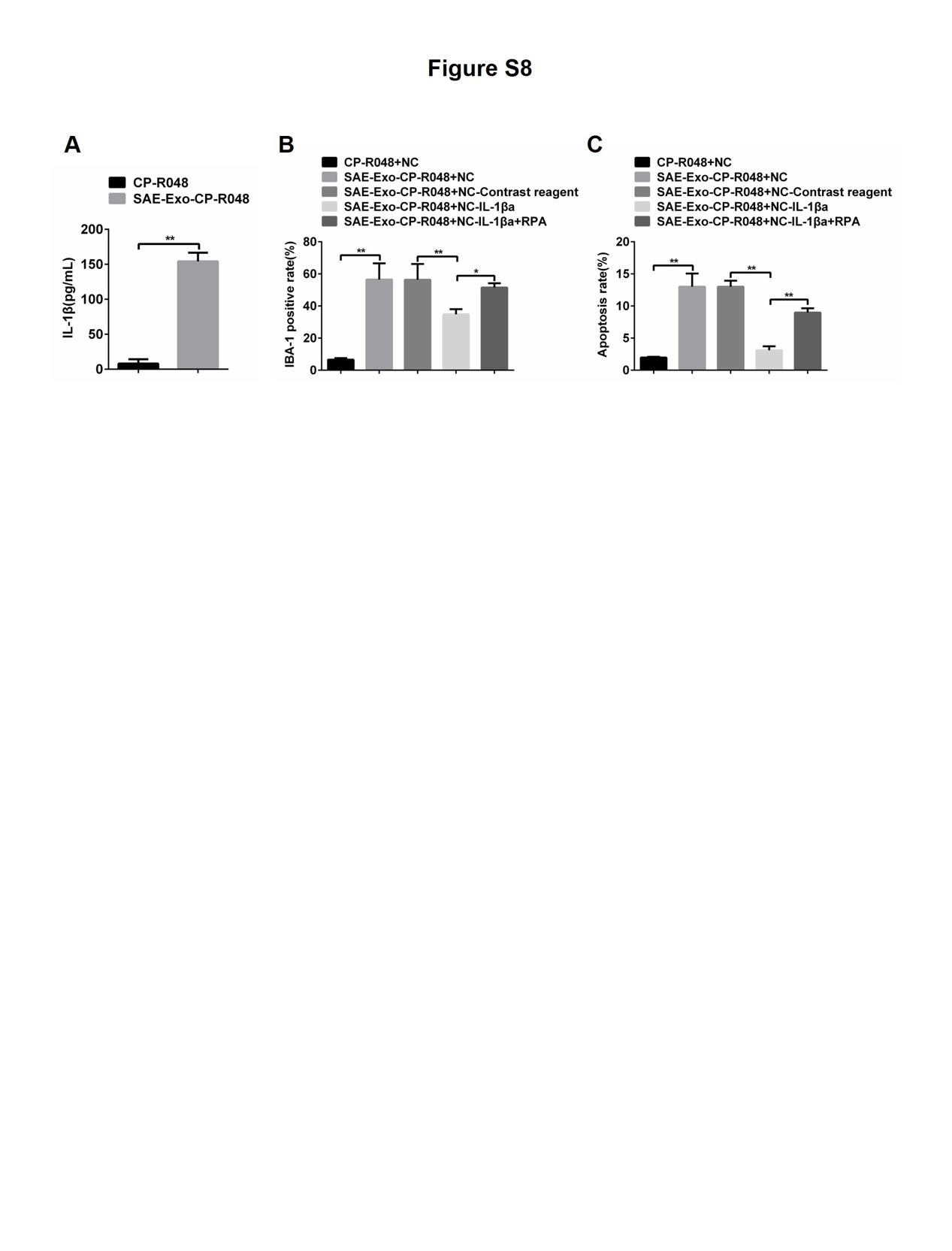


**Figure S8 Neuron injury detection in autophagy activator or IL-1β antagonist-exposed cells. (A)** Firstly, CO-R048 cells were treated with intestinal epithelial cell exosome from Sham and SAE rats. The supernatants were collected for the incubation of H19-7 cells. Then the levels of IL-1β was detected by ELISA kits. **(B)** Quantitative analysis of IBA-1-positive cells in H19-7 cells. **(C)** Quantitative analysis of the proportion of apoptotic cells in the above cells. *P<0.05. **P<0.01.
